# Stories From the Dendritic Cell Guardhouse

**DOI:** 10.3389/fimmu.2019.02880

**Published:** 2019-12-11

**Authors:** J. Kenneth Hoober, Laura L. Eggink, Robert Cote

**Affiliations:** Susavion Biosciences, Inc., Tempe, AZ, United States

**Keywords:** CLEC10A, DEC205, dectin-1, mannose receptor, endocytosis, calcium, immunosuppression, anergy

## Abstract

Phagocytic cells [dendritic cells (DCs), macrophages, monocytes, neutrophils, and mast cells] utilize C-type (Ca^2+^-dependent) lectin-like (CLEC) receptors to identify and internalize pathogens or danger signals. As monitors of environmental imbalances, CLEC receptors are particularly important in the function of DCs. Activation of the immune system requires, in sequence, presentation of antigen to the T cell receptor (TCR) by DCs, interaction of co-stimulatory factors such as CD40/80/86 on DCs with CD40L and CD28 on T cells, and production of IL-12 and/or IFN-α/β to amplify T cell differentiation and expansion. Without this sequence of events within an inflammatory environment, or in a different order, antigen-specific T cells become unresponsive, are deleted or become regulatory T cells. Thus, the mode by which CLEC receptors on DCs are engaged can either elicit activation of T cells to achieve an immune response or induce tolerance. This minireview illustrates these aspects with Dectin-1, DEC205, the mannose receptor and CLEC10A as examples.

## Introduction

Carbohydrate-recognition domains (CRDs), or more specifically C-type lectin-like domains (CTLDs), have evolved to generate a myriad of surface proteins that allow cells to monitor their environment and sense danger. The repertoire of glycan and non-glycan ligands that these receptors recognize is vast and internalization of the ligand begins the process of presentation of antigen to T cells and generation of an immune response. A striking feature of DCs is their 3- to 4-fold greater surface area to volume ratio than that of macrophages or lymphocytes, which provides a greater “sweep volume” for DCs to scan their environment (1). The larger surface area allows contact with ~5,000 T cells per hour, with an average contact duration of about 3 min. The minimum dwell time for TCR triggering is 2 s, and the half-life of contact between antigen-presenting cells (APCs) and T cells is 120 s, which is well within the 3-min window (2). The number of different CLEC receptors expressed by myeloid cells mark them as truly sentinels of the immune system (3–5). But these receptors can also cause DCs to become “tolerogenic” or induce unresponsiveness (anergy) in T cells (6, 7).

## Discrimination of Ligands

CLEC receptors, by forming clusters, distinguish between ligands of different sizes. Whereas, a single sugar generally binds with relatively low affinity (millimolar to micromolar range), multivalent ligands on the surface of pathogens bind with several orders of magnitude greater avidity (nanomolar to picomolar). Thus, the combination of CLEC receptors, which are often multimeric (8), and multivalent ligands provide a highly sensitive detection system. A revealing example of the role of these factors is recognition and discrimination of ß-glucans by Dectin-1 (CLEC7A, CD369) (9). Large ß-glucan polymers such as yeast cell walls induce formation of extensive clusters of Dectin-1 along the particle surface that exclude the cell membrane phosphatases, CD45 and CD148. Consequently, the hemi-immunoreceptor tyrosine-based activation motif (hemITAM, i.e., a single YxxL/F motif with an upstream triacidic sequence) in the cytoplasmic domain of Dectin-1 initiates signals through the kinases Src and Syk (9). This motif is also present in the cytoplasmic domain of other C-type lectin receptors (3, 4, 10) and is in contrast to the two immunoreceptor tyrosine-based activation motifs in tandem that describe an ITAM (YxxI/Lx_6−12_YxxI/L) (3, 4). The phagocytic process involves massive reorganization of cellular membranes, coordinated by the actin cytoskeleton (11), which also generates reactive oxygen species by activation of NADPH oxidase in the plasma membrane (12) to kill the pathogen. Digestive enzymes are inserted into an acidified endolysosomal system to degrade the invader, and degradation products are then presented in MHC class I or class II complexes on the cell surface. The extensive structural changes of the cytoskeleton and plasma membrane required for phagocytosis are dependent on an elevated cytosolic concentration of Ca^2+^ (13). In contrast, small, soluble oligosaccharides of ß-glucans such as laminarin bind only a few Dectin-1 molecules and result in a receptor cluster too small to exclude the phosphatases. Thus, phosphorylation of tyrosine in the hemITAMs is not sustained and signal transduction is minimized (9).

Phagocytic cells also recognize polymers such as mannan, a polysaccharide of mannose and glucose, that is present in the yeast cell wall and recognized by the mannose receptor C-type 1 (MRC1, MR, CLEC13D, CD206) (14, 15). MRC1 is a type I membrane protein that contains 8 CRDs in its external domain (15) and is expressed on macrophages and immature DCs (16–18). DCs that emerge under inflammatory conditions from monocytes (mo-DCs or infDCs) highly express MRC1 (19, 20). Glycoproteins that contain mannose, fucose, glucose, or N-acetyl glucosamine bind MRC1 (15) and are found in early endosomes after endocytosis (21). Whereas, MRC1 binds preferentially to single mannose residues, the type II protein receptor DC-SIGN [dendritic cell-specific intercellular adhesion molecule (ICAM)-3 grabbing non-integrin, CLEC4L, CD209] has a single CRD but the multimeric receptor preferentially binds high-mannose oligosaccharides (22). DC-SIGN is expressed by murine and human monocyte-derived immature and mature DCs (23–26) but not by DCs that differentiate from monocytes under the highly inflammatory conditions of ovarian ascites in humans (20). Other C-type, mannose-binding receptors are the type II receptors Dectin-2 (CLEC6A) and langerin (CLEC4K, CD207) (27), and C-type lectin DC immunoreceptor (DCIR, CLEC4A, CD367), which belongs to the Dectin-2 family and contains an immunoreceptor tyrosine-based inhibitory motif (ITIM) and sustains type I IFN (IFN-α/β) signaling through the transcriptional factor STAT3 (28).

Carcinoma cells richly express the Tn antigen (N-acetylgalactosamine [GalNAc]-*O*Ser/Thr) (29, 30), which binds to the type II GalNAc-specific, C-type lectin domain family 10 member A (CLEC10A, CD301, also designated the human macrophage galactose-type lectin, hMGL, or simply MGL) on DCs and macrophages (31–33). Two forms of MGL were identified in the mouse, mMGL1 (CD301a), which preferentially binds ligands containing terminal galactose, and mMGL2 (CD301b), which is specific for GalNAc (34). Subcutaneous injection of the Tn antigen into patients bearing a carcinoma tumor causes a delayed hypersensitive response, indicative of the presence of Tn-specific T cells (30). However, the single sugar ligand, as the Tn antigen, binds with relatively low affinity (K_D_ = 8–12 μM) to CLEC10A (33, 35). Linear glycopeptides (33, 35) or dendrimers built on a tri-lysine core (36–38), which contain 4–12 GalNAc residues, have K_D_ values of 50–100 nM, an indication of the effect of multivalency. These larger structures induced a strong Tn-specific T_H_2 and B-cell anti-tumoral antibody response with secretion of IL-4, IL-5, IL-13, and IL-10 (38). Kurze et al. (39) showed that expression of the Tn antigen is induced by tamoxifen, oxidative stress, and DNA damage in breast cancer tissues, which enhanced binding of CLEC10A (40), and was associated with improved outcome and survival.

Ligand constructs that include the Tn antigen take advantage of the sugar as the specific natural ligand for CLEC10A (38). However, a short peptide sequence has been found that mimics GalNAc and binds to CLEC10A with much higher avidity (41). This peptide is effective in suppressing ovarian ascites, which is an inflammatory environment that recruits monocytes that differentiate into DCs that express CLEC10A (41–43). The response to treatment of ovarian ascites in mice with this peptide demonstrated that not only the properties of the ligand but also the tumor environment and frequency of dosing determine whether co-stimulatory factors continue to maintain activity of T cells or DCs initiate tolerance (41).

CLECs often function in combination with toll-like receptor-2 (TLR-2) for detection of pathogens (36, 44, 45), which forms heterodimers with TLR1 or TLR6 to bind lipoteichoic acids and lipoproteins in the pathogen cell wall. The cytoplasmic domain of TLR2 binds the adapter proteins TIRAP and MyD88 and activates a signal transduction pathway that leads to NF-κB and AP-1 and eventual phagocytosis (46–48). van Vliet et al. (36) and Heger et al. (49) found that antibodies against MGL (CLEC10A) on human DCs did not induce release of IL-10 or IL-12 but stimulated release of IL-10 and TNF-α upon addition of TLR ligands (Pam3CysK4 for TLR2, LPS for TLR4, or R848 for TLR7/8).

Subsets of human DCs express distinct patterns of CLEC receptors. Dectin-1, DEC205 (CLEC13B, LY75, CD205), and DCIR are highly expressed on CD141^+^, CD1c^+^, and CD16^+^ subtypes (50). As analyzed by single-cell RNA sequencing, CD141^+^ DCs (DC1) express, among other distinctive genes, the unique marker CLEC9A, whereas CD1c^+^ DCs (DC2A and DC2B) express the unique marker CLEC10A (49, 51, 52). Mo-DCs in the inflammatory ascites environment are DC2-type in which CLEC10A but not CLEC9A is expressed (42).

## Endocytic Receptors

Immature DCs actively internalize surrounding material by “non-specific” macropinocytosis and “specific” receptor-mediated endocytosis and phagocytosis (53, 54). A specific “endocytosis” motif, the tyrosine-containing sequence YENFY in MGL (CLEC10A), YKSL in DC-SIGN, FENTLY in MRC1, and FSSVRY in DEC205 were identified in the cytoplasmic domain of several CLEC receptors that are similar to the hemITAM (15, 24, 55). As with other regulatory motifs, phosphorylation of the tyrosine residue in the motif is required for activity. Valladeau et al. (56) described an asialoglycoprotein receptor, homologous to the hepatic ASGPR-1 (57), that is expressed by DCs and designated DC-ASGPR. Similarly to CLEC10A, DC-ASGPR undergoes ligand-induced endocytosis with a t_½_ of 5–8 min at 37°C and delivers bound antibodies to early endosomes (55, 56, 58). The short isoform of DC-ASGPR is identical to the sequence of human CLEC10A, with the exception of a three-amino acid deletion near the base of the CRD (56). The long isoform has an insertion of 27 amino acids in the membrane proximal region of the extracellular CRD. Higashi et al. (25) described a total of seven isoforms of this receptor in humans, which are splicing variants from a single gene, and all contain the YENF endocytosis motif in the cytoplasmic domain. Overall endocytic activity decreases as DCs mature in response to inflammatory conditions (53, 54), including expression of MRC1 (54) and CLEC10A (49). However, expression of DEC205 was upregulated in the mouse and continued to capture antigens as DCs matured (54).

Within the cell, a second level of discrimination occurs. Internalization, degradation of the pathogen within the endosomal/lysosomal vacuolar pathway, and antigen presentation on the cell surface may require an extended period of time, depending on the ligand (21, 58–60). Large glycoproteins can be trapped in early endosomes and progress slowly through the vacuolar system for digestion and presentation on MHC class II complexes. In contrast, small glycopeptides enter the vacuolar system rapidly but also enter the cytosol and are cross-presented via MHC class I complexes to CD8^+^ T cells. Mo-DCs in inflamed tissues efficiently cross-present small antigens (10- and 26-mer peptides) to CD8^+^ T cells and stimulate CD8^+^ T cell proliferation and secretion of IFN-γ with the help of CD4^+^ T cells (42). A modified MUC1-derived glycopeptide bearing multiple Tn antigens was internalized by CLEC10A expressed by DCs and processed through MHC class I and II pathways but was still detectable in these compartments 24 h later (58). Similar to endocytosis by MRC1, a larger MUC1 glycoprotein was confined to the endosomal compartments and was not processed through the MHC pathways.

Although Dectin-1 is an exception (61), CLEC receptors generally require 2–4 Ca^2+^ ions in the CRD to bind a ligand (22, 33, 57). Endocytosis of these receptors therefore transfers bound Ca^2+^ into the endosome along with Ca^2+^ in extracellular fluid. The large concentration gradient of Ca^2+^ from ~1 to 2 mM outside the cell to 100 nM in the cytosol drives a flux across the endosomal membrane (32). As a “second messenger,” Ca^2+^ activates metabolic pathways through regulatory proteins (62–64). Activation of protein kinase C and calmodulin-regulated networks of kinases and phosphatases such as calmodulin-dependent kinase II and calcineurin occurs within minutes. The initial increase in Ca^2+^ may be augmented by activation of phospholipase C, which generates inositol trisphosphate, the signal for release of Ca^2+^ from the endoplasmic reticulum (65, 66).

## Induction of Tolerance by CLEC Receptors

In 1987 Jenkins and Schwartz presented evidence that antigen-specific T cells cultured *in vitro* with splenocytes, which were coupled to antigen through a carbodiimide derivative, became unresponsive within several hours (67). The effect appeared within 2 h, was essentially complete by 16 h and lasted more than a week. These results were confirmed with *in vivo* studies and indicated that the carbodiimide derivative inactivated a factor on the antigen-presenting cells that was required to sustain T cell activity. Some years later, Steinman and colleagues observed that when the C-type lectin receptor DEC205 on DCs in mice was engaged by subcutaneous injection of an anti-DEC205 antibody-antigen conjugate and T cells were isolated 2 days later and challenged with the antigen, activation of antigen-specific T cells was demonstrated by the release of IFN-γ and IL-2 and a proliferative response. However, when challenged 1 week later, T cells were unresponsive (17). Injection of an antibody agonist of CD40, a co-stimulatory protein expressed by DCs, sustained activation of T cells. Thus, in the absence of co-stimulation, presentation of antigen by “steady-state” or immature DCs led to transient activation of antigen-specific T cells followed by T cell deletion and anergy in surviving cells (17, 68).

The discovery of tolerance by delivery of antigens through DEC205 to immature DCs led to an extensive line of research into treatments for autoimmune diseases (69–71). In particular, when the myelin oligodendrocyte glycoprotein (MOG) was coupled to an antibody specific for DEC205 and injected intravenously into mice, the symptoms of experimental allergic encephalomyelitis (EAE), a model system for muscular dystrophy, were drastically suppressed (69). However, intravenous injection of MOG_35−55_, a major autoimmune epitope of the glycoprotein, alone also suppressed the symptoms of EAE (72). Definitive evidence for the role of DEC205 in EAE tolerance were experiments in which anti-DEC205/MOG was injected subcutaneously (17, 70) or intraperitoneally (71). This treatment also elevated the number of IL-10-secreting Treg cells, which was dependent on the transcription factor Hopx (71). Moreover, antigen-loaded DCs that migrate to draining lymph nodes have a superior ability to generate Treg cells to support the tolerogenic state (70, 73). Similar results were obtained in a mouse model of rheumatoid arthritis with a proteoglycan conjugated to an antibody against DEC205 (74).

DEC205 is a type 1 protein receptor that contains 10 CRDs (15) and has a short cytoplasmic tail with an endocytic motif similar to MRC1 (15, 24, 75). DEC205 does not bind a sugar but is a receptor for CpG-rich oligonucleotides (76, 77) The filamentous bacteriophage fd, whose single-stranded DNA is rich in CpG, binds to DEC205 and effectively delivers antigens to late endosomes or lysosomes (77). As with MRC1 (21), DEC205 recycles back to the cell surface within 1 h (75). In humans, mature DCs down-regulate MRC1 and DEC205-mediated endocytosis (54, 78). DEC205 is over-expressed in high-grade serous ovarian tumors as compared with low malignant potential tumors or normal tissues (79). A fully humanized monoclonal antibody against DEC205, when cross-linked to a cleavable maytansinoid derivative that disrupts microtubule function, targeted the receptor on tumor cells and was an effective anticancer agent (80).

An antibody against MRC1 on immature mo-DCs induced maturation of the cells as indicated by upregulation of CD80/83/86 but also increased secretion of IL-10 and decreased secretion of IL-12 (18). As with DEC205, T cells cultured with these DCs initially had a proliferative response but then became unresponsive to challenge. An aggregate of lipoarabinomannans from *Mycobacterium bovis* or *M. tuberculosis*, capped with mannose, bound to MRC1 and inhibited release of IL-12, a key factor in development of the T_H_1 response by T cells (81). MRC1 brings the cancer-related, highly glycosylated protein MUC1 into early endosomes when taken up by DCs but is recycled to the cell surface with some of the ligand still attached (21). MUC1 that dissociated from the receptor remained undegraded for more than 24 h within early endosomes (58). The inertness or slow processing of MUCI results when the sites of cleavage by lysosomal cathepsin L are blocked by glycosylation, and thus presentation to CD4^+^ T cells lags far behind the endocytic event (82). In apparent contrast, MUC1 coupled to mannan and then oxidized with sodium periodate to introduce aldehyde groups rapidly entered macrophages through MRC1, was processed in the cytosol and activated CD8^+^ T cells through the MHC class I pathway (83, 84).

Targeting DC-ASGPR (CLEC10A) on mo-DCs with antigen-conjugated antibodies induced antigen-specific CD4^+^ T cells to produce the cytokine, IL-10 (85). Intradermal injection of these conjugates into non-human primates also elevated IL-10 and reduced IFN-γ levels in blood. Further studies showed that binding of an antibody to DC-ASGPR induced maturation of DCs, indicated by expression of CD86, and activated a Src/Syk signal transduction pathway that includes PLCγ2, ERK1/2, p90RSK, and CREB, which led to expression of IL-10 (86). The increased secretion of IL-10 but decreased secretion of IFN-γ and IL-12 are indicative of a tolerogenic profile.

Within the reciprocal relationship between the anti-inflammatory IL-10 and pro-inflammatory IL-12 (87), IL-10 is produced by APCs that are activated by an increase in intracellular Ca^2+^ (88–92). Endocytosis of pathogens by CLECs expressed by these cells increased intracellular Ca^2+^, which can lead to expression of the *IL10* gene via the signal transduction pathway described above by Gu et al. (86). Phosphorylation of the transcriptional factor CREB by calmodulin-dependent protein kinases mediates the response to Ca^2+^ and production of IL-10 (93). In contrast, *IL12* expression is promoted by low intracellular Ca^2+^ concentrations and regulated by NF-κB (89, 94, 95).

## Regulation of T Cell Anergy and Exhaustion

Extensive evidence has been obtained that presentation by DCs of extrinsic antigen alone to T cells is insufficient to maintain T cell activity and leads to tolerance. Activation of T cells is induced by mature DCs, but either absence of a co-stimulatory signal or inability to process an antigen allows an inhibitory system in T cells to become dominant, which leads to a state of anergy (6, 7, 96). Heissmeyer et al. (97) showed that a sustained elevated Ca^2+^ signaling induces a state of unresponsiveness in T cells by calcineurin-mediated degradation of PLC γ1 and PKCθ. PLC γ1 activity leads to activation of NFAT, whereas PKCθ activity is required for activation of AP-1, which in combination with NFAT induces expression of activation genes (98). Without AP-1, NFAT locks in an inhibitory pattern of gene transcription (96–98). A more lengthy process, described as “exhaustion,” is associated with loss of effector T cell function and altered transcriptional patterns (99). Whereas, anergy is achieved within a few days, development of exhaustion requires weeks. The exhausted state can be overcome by checkpoint blockade (100).

Functional presentation of digestion products to T cells requires three signals. Sckisel et al. (101) stated that “primary T cell activation is tightly regulated and requires three signals in sequence: signal 1, where TCR recognition of cognate antigen in the context of major histocompatibility complex (MHC) restriction occurs; signal 2, involving binding of costimulatory molecules; and signal 3, where cytokine ‘instructions' direct and amplify T cell differentiation and expansion.” Low density (i.e., low concentrations or poor affinity) of an antigen can lead to anergy; higher concentrations of the antigen can maintain T cell activation (102). The inflammatory stimulus (signal 3) occurs via secretion by DCs of cytokines such as IL-12, the principal cytokine for a T_H_1 response, or type I interferon (IFN-α/β) (43, 103). Secretion of the type I interferons is an expression of danger signals emitted by stressed cells that provide the essential context for an immune response, without which T cells receive a tolerogenic signal from DCs (104–106). Tolerance that results from clonal deletion of T cells to which antigens are presented by “steady state” DCs is a major factor in prevention of autoimmunity (104).

A major characteristic of immature DCs is their capacity as sentinels to scan their environment, perform receptor-mediated endocytosis and phagocytosis, with subsequent maturation and interaction with T cells. CLEC receptors are intimately involved with instructions, described above, given to T cells that determine their course of action, whether destruction of tumor cells or, by different mechanisms, anergy or tolerance. Along with understanding conformational changes within the CRD induced by ligand binding (107), the signals that lead to these functional consequences are becoming clear.

## Addendum

Taylor and Drickamer (108) provided an up-to-date review of mammalian sugar-binding receptors. A complete catalog of CLEC receptors is at the web site for the Imperial College London^1^.

## Author Contributions

This manuscript was written by JH with editorial and literature assistance from LE and RC. All authors approved the version for submission.

### Conflict of Interest

LE and JH are co-founders of Susavion Biosciences, Inc., and inventors of intellectual property that has been assigned to Susavion Biosciences, in which these authors hold shares. RC is an independent consultant for Susavion Biosciences.

## Footnotes

^1^http://www.imperial.ac.uk/research/animallectins/ctld/default.html http://www.imperial.ac.uk/research/animallectins/ctld/mammals/domain.html http://www.imperial.ac.uk/research/animallectins/ctld/mammals/mammals.html
